# A Multi-Epitope Protein for High-Performance Serodiagnosis of Chronic Chagas Disease in ELISA and Lateral Flow Platforms

**DOI:** 10.3390/ijms25189811

**Published:** 2024-09-11

**Authors:** Evandro R. Dias, Andressa M. Durans, Barbara B. Succar, Luiz André L. T. Pinto, Guilherme C. Lechuga, Mariana G. Miguez, Janaina Figueira-Mansur, Ana P. C. Argondizzo, Aline R. Bernardo, Rafaela L. Diniz, Gabriela S. Esteves, Edimilson D. Silva, Carlos M. Morel, José Borges-Pereira, Salvatore G. De-Simone, Angela C. V. Junqueira, David William Provance

**Affiliations:** 1 Center for Technological Development in Health, National Institute of Science and Technology for Innovation in Neglected Population Diseases, Oswald Cruz Foundation, Rio de Janeiro 21040-361, RJ, Brazil; evandrodias@aluno.fiocruz.br (E.R.D.); andressa.durans@fiocruz.br (A.M.D.); barbara.barbosa@fiocruz.br (B.B.S.); luiz.teixeira@int.gov.br (L.A.L.T.P.); guilherme.curty@fiocruz.br (G.C.L.); carlos.morel@fiocruz.br (C.M.M.); salvatore.simone@fiocruz.br (S.G.D.-S.); 2 Tropical Medicine Graduate Program (Stricto Sensu), Oswaldo Cruz Institute, Oswald Cruz Foundation, Rio de Janeiro 21040-361, RJ, Brazil; junqueir@ioc.fiocruz.br; 3 Laboratório de Epidemiologia e Sistemática Molecular, Oswaldo Cruz Institute, Oswald Cruz Foundation, Rio de Janeiro 21040-361, RJ, Brazil; 4 Laboratório de Doenças Parasitarias, Oswaldo Cruz Institute, Oswald Cruz Foundation, Rio de Janeiro 21040-361, RJ, Brazil; borges@ioc.fiocruz.br; 5 Laboratório de Tecnologia Recombinante, Institute of Technology in Immunobiology, Oswald Cruz Foundation, Rio de Janeiro 21040-361, RJ, Brazil; mariana.garcia@bio.fiocruz.br (M.G.M.); janaina.mansur@bio.fiocruz.br (J.F.-M.); acorrea@bio.fiocruz.br (A.P.C.A.); gabriela@bio.fiocruz.br (G.S.E.); 6 Laboratório de Reativos em Diagnóstico, Institute of Technology in Immunobiology, Rio de Janeiro 21040-361, RJ, Brazil; aline.bernardo@bio.fiocruz.br (A.R.B.); rafaela.diniz@fiocruz.br (R.L.D.); edmilson@bio.fiocruz.br (E.D.S.); 7 Science and Biotechnology Graduate Program, Department of Molecular and Cellular Biology, Biology Institute, Fluminense Federal University, Niterói 24020-141, RJ, Brazil

**Keywords:** Chagas disease, diagnostics, ELISA, lateral flow tests, synthetic protein, *T. cruzi*

## Abstract

We developed a protein to rapidly and accurately diagnose Chagas disease, a life-threatening illness identified by the WHO as a critical worldwide public health risk. Limitations in present day serological tests are complicating the current health situation and contributing to most infected persons being unaware of their condition and therefore untreated. To improve diagnostic testing, we developed an immunological mimic of the etiological agent, *Trypanosoma cruzi*, by combining ten pathogen-specific epitopes within the beta-barrel protein structure of Thermal Green Protein. The resulting multi-epitope protein, DxCruziV3, displayed high specificity and sensitivity as the antibody capture reagent in an ELISA platform with an analytical sensitivity that exceeds WHO recommendations. Within an immunochromatographic platform, DxCruziV3 showed excellent performance for the point of application diagnosis in a region endemic for multiple diseases, the municipality of Barcelos in the state of Amazonas, Brazil. In total, 167 individuals were rapidly tested using whole blood from a finger stick. As recommended by the Brazilian Ministry of Health, venous blood samples were laboratory tested by conventional assays for comparison. Test results suggest utilizing DxCruziV3 in different assay platforms can confidently diagnose chronic infections by *T. cruzi*. Rapid and more accurate results will benefit everyone but will have the most noticeable impact in resource-limited rural areas where the disease is endemic.

## 1. Introduction

Chagas disease, also known as American trypanosomiasis, is a potentially life-threatening illness caused by the protozoan parasite *Trypanosoma cruzi* [1]. Endemic to Latin America, this disease was primarily transmitted through the feces of triatomine bugs until the 1990s when programs were implemented to control *Triatoma infestans*, the species mostly responsible for infections inside homes [2,3].

Today, many people in tropical areas remain at risk from other species of triatomine in remote work areas or from drinking *T. cruzi*-contaminated fresh unpasteurized fruit juices [4,5]. Other modes of transmission include blood transfusions, organ transplants, and from mother to child during pregnancy, which can occur in non-endemic regions due to the migration of infected individuals [6,7,8]. Overall, up to 75 million people are at risk of infection globally [9].

Of the 6 to 7 million people worldwide that are estimated to be infected with *T. cruzi,* only around 10% are aware of their condition [9]. Chagas disease progresses through two phases: acute and chronic [10,11]. Because the acute phase is often asymptomatic or presents mild symptoms, the disease is frequently undiagnosed at this stage. Untreated, the disease progresses to the chronic phase, which can remain silent for decades. In about 30% of chronically infected individuals, the disease can cause cardiac, digestive, or neurological complications. The most severe form of Chagas disease involves cardiomyopathy, leading to heart failure, arrhythmias, and sudden death [12,13,14]. Gastrointestinal manifestations include megaesophagus and megacolon, which can cause severe digestive issues [15,16].

Although serological diagnosis plays an essential role in identifying persons with a chronic infection for managing Chagas disease, at least two different serological tests are recommended for a definitive diagnosis. Cross-reactivity with antibodies generated against other parasitic infections has been a major issue for specificity. Moreover, many commercial tests show variability in sensitivity according to the geographical origins of an individual [17,18]. Our solution focuses on epitopes specific to *T. cruzi* and grouping them together through incorporation into the loops of the beta-barrel structure from green fluorescent protein [19]. While two resulting recombinant proteins—DxCruziV1 and V2—were recovered in inclusion bodies, in-house ELISAs showed that each displayed nearly perfect specificity and excellent sensitivity using two overlapping sets of ten epitopes.

To improve accessibility, affordability, and speed to screen populations in resource-limited areas where the disease burden is highest, the Brazilian Unified Health System suggests that using a point-of-care diagnostic test could be a viable alternative for diagnosing chronic Chagas disease [20]. A rapid test would also be strongly favored for use during prenatal examinations and during delivery when Chagas disease is suspected in the pregnant woman [21].

To meet the government’s goals, we exchanged the beta-barrel to a fully synthetic construct, Thermal Green Protein [22], with the intention of improving the production of soluble protein for use in both ELISA and lateral flow platforms. Previously characterized patient sera and international biological standards for Chagas disease were used to evaluate the performance of the resulting protein, DxCruziV3, in an ELISA platform. Its utility in a lateral flow platform was also evaluated by comparing field results to laboratory-based assays.

## 2. Results

### 2.1. Design and Production of DxCruziV3

The third version of DxCruzi maintained the epitopes contained in the second version with an extension of epitope 6 from TSSA by four amino acids on the amino terminus (Table 1), which is restricted to the *T. cruzi* Discrete Typing Units TcII, V, and VI found predominately in the southern cone [23]. Epitopes 3, 4, and 8 represent different permeations of a tandemly arranged repeat. The major difference among versions was a switch in the beta-barrel sequences to those of Thermal Green Protein (TGP). As a fully synthetic construct, the potential presence of pre-existing antibodies in patient serum would be further reduced compared to eGFP. In addition, TGP contains specific amino acid changes in the beta sheets of the barrel structure that diminish aggregation and improve stability, all desirable characteristics for an immunological mimic of a pathogen for serodiagnosis. As a first prediction for the potential to successfully produce protein, the projected primary sequence was submitted to the I-TASSER server [24,25].

**Figure 1:**
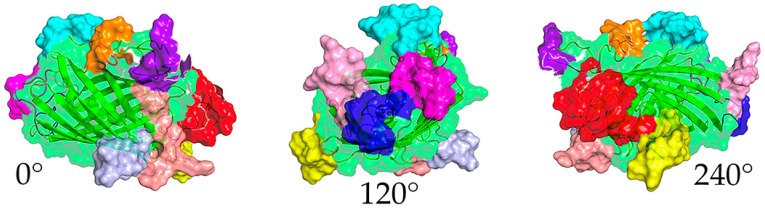
Computer model of DxCruziV3. Views at 0°, 120°, and 240° of the predicted structure. The retained beta-barrel is displayed with a ribbon for the primary sequence and a transparent surface (green). Epitopes are shown with a solid surface in the colors listed in Table 1. Projections obtained with PyMol software (V2.5.5, Schrödinger, NY, NY, USA).

A projection of three views of the computationally determined tertiary structure displayed a fully intact beta-barrel structure that suggested that the insertion of the epitope sequence did not disrupt its formation (Figure 1). This was confirmed by the robust expression of DxCruziV3 using the pET28a plasmid based on T7 RNA polymerase [36], which distributed to both the soluble fraction (~15%) and inclusion bodies (~85%). Due to the greater yield from inclusion bodies, insoluble DxCruziV3 (V3_ib_) was initially purified by IMAC after solubilization in 4 M urea (Figure S3). To reduce the urea concentration, samples were dialyzed against PBS to a final concentration of 1 M.

### 2.2. Performance of DxCruziV3 in ELISAs

**Figure 2:**
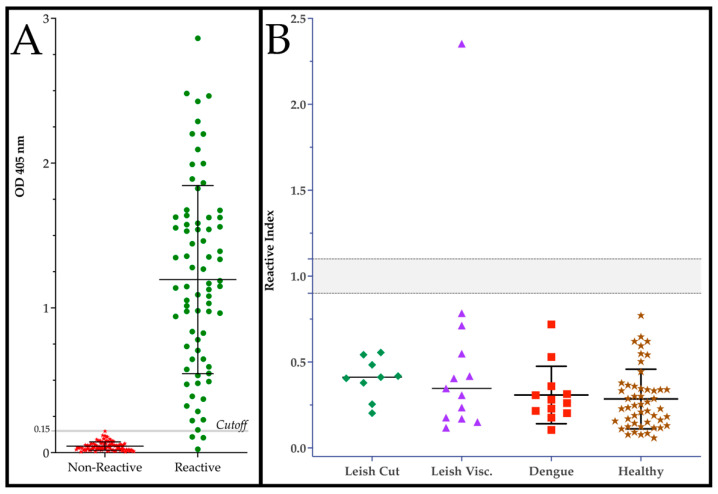
Performance of DxCruziV3_ib_ in an ELISA platform. (**A**) Measured reactivity (OD_405_) for individual samples of patient sera diagnosed *T. cruzi* infected (reactive; n = 75) or not (non-reactive; n = 104) by LACENs in the Brazilian states of Ceará, Sergipe, Paraíba, and Maranhão. Gray line marks cutoff. (**B**) Cross-reactivity against sera from patients diagnosed with cutaneous or visceral Leishmaniosis, and dengue as well as non-specific reactivity with samples from healthy blood donors (HEMORIO, Rio de Janeiro, RJ, Brazil). Gray line demarks borderline reactivity (±10%).

In-house ELISAs were generated using 200 ng of purified V3_ib_ maintained in 1 M urea to sensitize each well followed by an incubation with patient serum diluted 1:200. As can be seen in Figure 2A, the detection of human IgG showed excellent performance for differentiating reactive, infected from non-reactive, not infected individuals. An ROC analysis calculated an area under the curve of 0.9892 with a standard error of 0.0096 and a *p* value < 0.0001. With a cutoff of 0.153 OD_450_ and likelihood ratio of 98.6, the ELISA presented a sensitivity of 96.0% and a specificity of 100%. This translates to a positive likelihood ratio of 98.8% and a negative likelihood ratio of 95.3%. Using the cutoff, we determined the reactivity index for the detection of cross-reactivity and non-specific reactivity (Figure 2B). Using patient serum infected with dengue or *Leishmania* spp., only one of fifty-seven samples showed a reactivity above 1, which was not previously diagnosed for Chagas disease. Importantly, none of the healthy samples showed reactivity.

### 2.3. Analytical and Geographical Sensitivity of DxCruziV3

**Figure 3:**
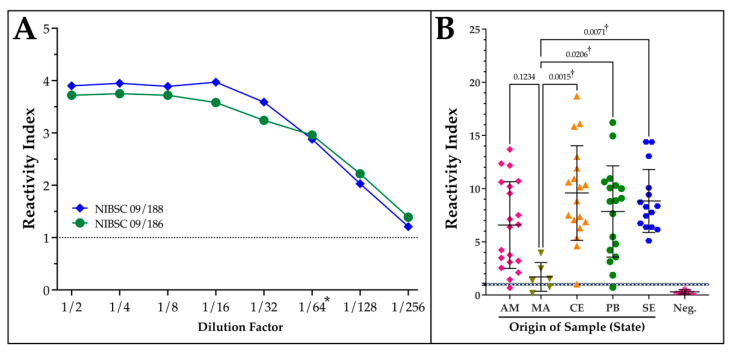
Analytical sensitivity and influence of geographical origin of patient sample on ELISA performance. (**A**) In-house ELISAs generated with 200 ng of V3_ib_ were exposed to a two-fold dilution series (1:2 to 1:256) of the two WHO International Biological Standards NIBSC 09/188 (TcI) and NIBSC 09/186 (TcII). Bound human antibodies were detected with a horseradish peroxidase labeled secondary. (**B**) Reactivity index calculated for patient samples originating from different states of Brazil displaying the *p* value from an ANOVA analysis with a Kruskal–Wallis test with borderline reactivity marked by dotted line. AM—Amazonas; MA—Maranhão; CE—Ceará; PB—Paraíba; SE—Sergipe. Controls (Neg.) represent non-reactive samples screened for Chagas disease. * Minimum WHO recommended dilution. ^†^ Significant difference.

The analytical sensitivity of the in-house ELISA assay was assessed by the application of the two WHO International Biological Standards for Chagas disease that represent patient sera from areas predominantly occupied by *T. cruzi* DTUs TcI (NIBSC 09/188; Mexico) and TcII (NIBSC 09/186; Brazil/Chile) over a 2-fold serial dilution. After converting absorbance values to a reactivity index (RI), the use of V3_ib_ as the antibody capture reagent resulted in an assay that exceeds the minimum recommended dilution value by a factor of 4 (Figure 3A). One aspect of the two standards is to be able to evaluate the potential for differences in sensitivity related to the geographical origin of the patient samples that could reflect the distribution of different subtypes of *T. cruzi*. To extend this to our panel of patient serum that represents populations across northern Brazil, the RI was calculated and plotted separately based on geography (Figure 3B). Following an ANOVA statistical analysis with a Kruskal–Willis test, the performance of the ELISA was significantly different in samples from the state of MA to the three more eastern states but not AM.

### 2.4. Lateral Flow Immunochromatographic Prototypes

The high level of sensitivity observed with V3_ib_ encouraged us to develop a lateral flow assay (LFA), which in general is considered to present lower sensitivities than an ELISA platform. A single lot of 300 LFAs was produced with DxCruziV3 purified from inclusion bodies. Two different flows were utilized to apply the control line of Protein A (0.5 µL/cm) and test lines of V3_ib_ (1.5 µL/cm) to achieve 200 ng and 450 ng per strip, respectively. Over the course of evaluating buffer compositions, the primary target performance profile was to eliminate the appearance of any coloration of the test line when non-reactive sera were applied. After finalizing the conditions for the V3_ib_-LFA, 100% sensitivity was confirmed using a pool of low-titer Chagasic patient sera and another with a high titer (Figure S4). Specificity was evaluated with serum pools with sera from patients with cutaneous leishimaniose, visceral leishimaniose, and dengue as well as healthy individuals and persons diagnosed as negative for chronic Chagas disease.

**Figure 4:**
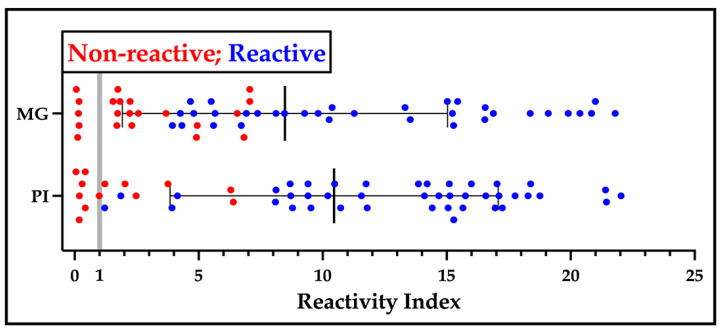
Performance of sV3-LFA in relation to V3_ib_-ELISA. Serum samples collected in the states of Minas Gerais (MG, BR) in 2022 and Piauí (PI, BR) in 1999 were applied (5 µL) to the sV3-LFA and defined as reactive (blue) or non-reactive (red). The samples were also analyzed by the V3_ib_, in-house ELISA (1:200 dilution) and their reactivity index calculated with the borderline reactivity marked by vertical gray line.

Independently, Bio-Manguinhos prepared sV3 (Figure S5), which was used to generate a single lot of LFAs. Internal quality control assays determined a sensitivity of 96% and a specificity of 100% (Table S1). A more extensive comparison was made with a panel of patient serum collected in the states of Minas Gerais and Piauí in 2022 and 1999, respectively. As can be seen in Figure 4, samples reactive in the sV3-LFA (blue) primarily displayed a higher reactivity index than non-reactive samples (red). Although some of the samples non-reactive by the LFA showed reactivity by ELISA, in no instance was there reactivity in the LFA and no reactivity by ELISA, suggesting that the LFA had the same high specificity but with a lower sensitivity than the ELISA platform. After the standardization phase, production lots of the two independent prototypes were evaluated by an application in the field followed by an in-depth laboratory analysis of the patient samples.

### 2.5. Performance of the LFA Prototypes

The municipality of Barcelos, AM, BR, was chosen as the geographical site for testing the LFAs due to its long-standing history as the site for studies on Chagas disease in the Amazon rainforest. A total of 16 communities within the municipality were represented by the recruited individuals. Overall, 43 of 167 participants (25.7%) were reactive by both LFAs. Two were reactive by V3_ib_-LFA and non-reactive by sV3-LFA along with two reactive by sV3-LFA and non-reactive by V3_ib_-LFA. The Nova Jerusalém community had the highest prevalence of reactive LFAs (19 of 52 reactive), followed by Mariuá (12 of 34 reactive) (Table S2). Men showed a higher prevalence than women (32 vs. 11), which represented 31% and 17% of the individuals of each sex, respectively. The vast majority were over the age of 40 years old (Table S3). In the lab, each correlate serum sample was subjected to a series of assays (Table 2). Initially, the two LFAs were repeated on field reactive samples along with a random subset of non-reactive samples due to a limitation in the number of tests produced in the first lot. In the place of 10 µL of whole blood, 5 µL of serum was applied under the controlled environment of the lab. One of the samples that was reactive in the field by V3_ib_-LFA was non-reactive in the lab, while three non-reactive samples showed a weak reactivity in the lab. For sV3-LFA, three samples that were reactive and one non-reactive in the field presented the opposite result in the lab.

The next assays were an indirect immunofluorescence (IFI) assay and the commercial ELISA kit from Bioclin in duplicate. In addition, the commercial ELISA kit from Wiener and in-house ELISAs utilizing V3_ib_ were employed. The results were defined as reactive (1) or non-reactive (0) and summed to group the results based on consistency between platforms. Of the 167 samples, 150 presented with a high level of agreement between the results from the assays. Within this group, both LFAs were calculated to have 100% sensitivity and specificity (Table 3). The ranking by decreasing sensitivity was the IF assay (93.8%) and then the ELISAs by Bioclin (90.6%), with V3_ib_ (87.5%), and by Wiener (71.9%). For specificity, the V3_ib_ and Wiener ELISAs showed very high performance, followed by the IF assays and the Bioclin ELISA. The remaining 17 samples were interrogated further to make a best evidence final call on the status of the patient through up to 18 different tests. The IFI, Bioclin, and Wiener assays were executed as instructed. A serial dilution of sera (1:50, 1:100, and 1:200) was applied to the V3_ib_-ELISA that showed a bifurcation of the samples between reactive and non-reactive (Figure S6). Ultimately, the calculations decreased for each assay although the effect was greater on sensitivity than specificity.

## 3. Discussion

While the connection between *T. cruzi* and Chagas disease was made by Carlos Chagas over 100 years ago [37], this parasite has been plaguing South America for thousands of years as evidenced by the detection of *T. cruzi* kinetoplast in the DNA of human mummies [38,39]. Today, it continues to be a predominantly silent life-threatening disease [40]. A troubling trend is the spread of Chagas beyond historic endemic regions with human migration and changes in climate [41]. With a goal of ending transmission, the 2030 WHO roadmap for neglected tropical diseases seeks to disrupt transmission routes and greatly increase treatment for afflicted individuals [42]. These goals cannot be met without an effective and economical diagnostic test.

It is well-known that current serological tests for Chagas disease do not meet the minimal target performance profile [43]. Nearly all display cross-reactivity with other diseases, especially *Leishmania* spp. [44,45], and have variances in sensitivity related to geography [18]. Both issues contribute to the need for two independent tests that can extend the time from sample collection to final diagnosis by several months. Recently, we reported on two multi-epitope proteins, DxCruziV1 and DxCruziV2, that could overcome these issues [19]. Built on the beta-barrel structure of eGFP, each displayed excellent qualities to serve as the antibody capture reagent in ELISAs.

Here, the beta-barrel was changed to that of TGP to yield DxCruziV3. The rapid folding and high stability of TGP was predicted to improve the solubility of the DxCruziV3 compared to the earlier versions. As a fully synthetic construct, the move to TFP was also anticipated to minimize background from non-specific antibody interactions. As expected, computer modeling showed a retention of the barrel structure with the epitopes exposed on the surface (Figure 1). To decisively demonstrate its potential to deliver high confidence diagnoses, DxCruziV3 was incorporated into ELISAs and rapid test platforms for interrogation with challenging patient samples and conditions. The resulting rapid test performances were extensively compared to three commercial tests.

As with the previous versions, DxCruziV3 proved to be easily expressed to high levels (>200 µg/mL) in bacteria with ~15% distributed in the soluble fraction. However, considering the yield and initial purity of protein in inclusion bodies, we decided to generate in-house ELISAs with V3_ib_. Using 200 ng/well, this could translate to nearly a million assays per liter of culture. To calculate specificity and sensitivity, we screened a panel of patient serum obtained from Brazilian state reference laboratories (LACENs) that had been diagnosed by the recommended protocol of two different assays followed by a third assay for divergent results. From 75 positive samples and 104 controls (Figure 2A), a specificity of 100% and a sensitivity of 96.0% were calculated by an ROC analysis (Table 1). Only a single sample from the panel of sera from patients diagnosed with either cutaneous Leishmaniosis, visceral Leishmaniosis, or dengue showed a reactivity index above one (Figure 2B). As the sample had not been previously screened for Chagas disease and these diseases are co-endemic, there is a good probability it could represent a dual infection rather than cross-reactivity. This potential highlights a difficulty related to reagent development in which most control patient serum samples are only defined for a single condition/disease. When that sample also represents the target profile, its inclusion can skew calculations toward a poorer performance.

While all of the serum samples in our panel for the ROC analysis have excellent provenance, they have been stored for years and subjected to an unknown number of freeze/thaws. For this reason, it was important to evaluate the V3_ib_-ELISA performance utilizing the two International Biological Standards for Chagas disease, which permit direct comparisons in analytical sensitivity between assays [46]. The initial flatness of the curve suggests that the available antibody binding sites were saturated up to a dilution of 1:16 (Figure 3A). Notably, a reactive index of greater than one was measurable through to a dilution of 1:256. This exceeds the minimum target dilution factor of 1:64 set by the WHO, which no commercial test in Brazil has achieved [47].

While no significant difference was observed between the two standards to suggest an influence of geographic origin on performance, significant differences were calculated between our samples from Maranhão compared to Ceará, Paraíba, and Sergipe but not Amazonas (Figure 3B). This result could reflect a greater loss of antibody titer during cold storage for low-titer samples, a small sample size, or could be restricted to the northern reaches of Brazil. It remains to be determined if the initial antibody titer influences retention during cold storage. Expanding the application of the assay to more regions of Brazil, other South American countries, and screening more recently obtained patient samples should conclusively demonstrate geographical differences. If it persists and compromises performance, a new group of epitopes could be organized to build a new reagent. Since the design of DxCruziV3, a complete atlas of epitopes in *T. cruzi* has been described that examined differences across the Americas [48].

It is clear our protein can aid with the creation of a high-performance ELISA that will help in managing Chagas disease. Of equal importance, DxCruziV3 can be used in a lateral flow test platform, which could rapidly deliver actionable results in remote endemic areas with an insufficient healthcare infrastructure to support laboratory-based tests but harbor a greater at-risk population. We had an opportunity to generate LFAs that utilized either sV3 or V3_ib_ completely independently of each other, which covered protein production, purification, and cassette assembly. Using pools of patient serum, the V3_ib_-LFA was assessed to be 100% specific and sensitive, while the post-production quality control results for sV3-LFA returned a specificity of 100% and a sensitivity of 96%. For a comparison of sensitivity between the LFA and ELISA platforms, sV3-LFA was investigated with two sera panels that had been analyzed by V3_ib_-ELISA. While one panel had been stored nearly 25 years, the results suggest that the LFA platform is less sensitive but retains a high specificity (Figure 4).

The municipality of Barcelos, AM, BR, was selected as the real-world test site to evaluate the LFA prototypes. This area has the archetype characteristics for the most challenging demographics and geographics: (1) it is a location where conventional tests have displayed low sensitivity; (2) it is endemic for multiple other diseases including malaria, dengue, and leishmaniosis; (3) many members of the community spend months on plantations that house *T. cruzi*-transmitting triatomines [49]; (4) the closest hospital is hours away by boat; (5) only two communities, Mariuá and Piloto, have an electrical grid while the others rely on diesel generators; (6) it is an archipelago that is difficult to access. To reach Barcelos, test kits were sent by plane from Rio de Janeiro, RJ, BR, to Manaus, AM, BR, and then traveled by boat for two days. While the boxed kits contained prototype LFAs sealed into individual foil packs with a silica gel pouch, no special effort was made during transport to avoid sunlight or the higher temperatures of springtime in the Amazon in the middle of a river.

In practice, the results of the LFAs at the point of application were clearly non-reactive or reactive with no failures to mark the control line. Forty-three individuals were suspected to be infected by *T. cruzi* and most reported working with piassava collection. To determine the validity of the results, venous blood samples were collected to obtain sera for laboratory analyses. These proved to provide less definitive results. In the end, six different assays were performed multiple times (Table 3). For 150 samples, there was a high level of agreement to define a sample as reactive or non-reactive, which suggested that the LFA results agreed to the highest possible extent with all the others. The remaining 17 samples required many other assays to reach a final call that still presented disagreements between assays.

Regardless, the final calculation showed a small decrease in specificity for the LFAs, V3_ib_-ELISA, and Wiener ELISA that remained above 95%. This most likely reflects the inclusion of many of the same epitopes in the capture reagent of the Wiener ELISA as in DxCruziV3 according to the bibliography cited with the product. For the Biolisa kit, the reagent to capture antibodies is not defined beyond a recombinant protein. With the IFI assay, the fixed parasites would contain natural proteins that present the highest number of possible antibody binding sites but also a higher number of non-specific sites that would decrease specificity [50]. The greatest impact was on sensitivity for all assays except the V3_ib_-ELISA. From the V3_ib_-ELISA assays on divergent samples, serial dilutions of 1:50, 1:100, and 1:200 revealed that the highest dilution could not definitively detect all reactive samples. In comparison, both of the lower dilutions showed a good differentiation and the results from two assays at 1:100 were used for the calculations.

Overall, the results suggest that this subgroup of reactive samples from the Amazon had anti-*T. cruzi* antibody titers near the limit of detection for the LFAs, and below the limits of the commercial tests. As the ELISAs from Wiener and Bioclin as well as the IFI assay use 10 µL of patient serum, attempting to increase sensitivity with a greater volume of sample would most likely be impractical. The volume used in the LFAs is also at a maximum, but the calculated sensitivity suggests that it is only slightly less than the V3_ib_-ELISA at a dilution of 1:100. This would be equivalent to a reactivity index approaching one, which converges with the analytical sensitivity of V3_ib_-ELISA at a dilution of 1:256 and suggests that both serodiagnostic platforms would satisfy WHO targets.

In the case of Chagas disease, we would argue that high specificity should take precedence over high sensitivity, more so if a low-cost test is available. False positives from a lower specificity would necessitate a need for a confirmation of the diagnosis before a medical decision since a subset would not require intervention. For Chagas disease, the available drugs require a long treatment period and have many undesirable side effects [51]. In contrast, a false negative would represent an undetectable antibody titer, which is also an end point for successful treatments [52,53]. Low titer would suggest a less active immune response to a less aggressive infection. Changes in an infection, as reflected by this immune response, could be detected by a yearly test for persons originating from or who had worked in endemic regions. Ultimately, a false negative result would still allow all positive individuals to receive immediate medical advice since there would be no doubt a reactive person is infected. Combined with a confirmatory ELISA with higher sensitivity, persons under medical supervision could be better accommodated.

While the absence of a definitive diagnosis for the patient samples gathered in Barcelos excludes an absolute calculation of specificity and sensitivity for the LFA protypes, it is clear that they exceed those of commercially available ELISA kits. Increasing the number of individuals tested and expanding the geographical areas to other regions of Brazil and other countries will demonstrate the level of confidence one can associate with the results. Chagas disease has been on ongoing challenge for developing an immunological reagent and we anticipate that the platform utilized to create versions of DxCruzi will have applications for other diseases and infectious agents.

## 4. Materials and Methods

### 4.1. Ethical Approval

All procedures were performed in accordance with The Code of Ethics of the World Medical Association (Declaration of Helsinki) for experiments involving humans and complied with relevant laws and institutional guidelines. Research protocols were pre-approved by the Research Ethics Committee of the Oswaldo Cruz Foundation for involving humans (CAAE: 52892216.8.0000.5248). In compliance with Resolution 466/12 of the National Health Council, participants were included after accepting and signing a consent form.

### 4.2. Serological Panels and Participant Profile

Serological samples were obtained from the Central Public Health Laboratories (LACENs) in the Brazilian states Ceará (CE), Maranhão (MA), Paraíba (PB), and Sergipe (SE) as well as samples from the city of Barcelos in the state of Amazonia (AM) (Figure S1). Samples from the LACENs were previously diagnosed for chronic Chagas disease by accepted protocols at the respective centers and reevaluated with Chagastest ELISA Rec. v3.0 (Wiener Lab, São Paulo, Brazil) and IFA-Chagas (Bio-Manguinhos, Rio de Janeiro, Brazil). The WHO International Biological Reference Standards—NIBSC 09/188 and NIBSC 09/186—that represent the TcI (Mexico) and TcII (Brazil and Chile) *T. cruzi* DTUs, respectively, were used to determine analytical sensitivity by a dilution series from 1:2 to 1:256 performed in triplicate [54].

In the municipality of Barcelos, AM, 167 participants were recruited that either lived in riverside communities or in boats temporarily anchored on the banks of the Negro River. In collaboration with local healthcare providers, residents were interviewed for their willingness to participate and history of frequenting plantations with piassava palm trees, a region reported to support the presence of triatomines [55]. In some instances, whole families participated that included a few minors (n = 7). Two participants did not respond to this question and thirty-one said they did not frequent the piassava extraction area. Inclusion of indigenous persons was graciously permitted by the Special Health District for Indians in upper Negro River.

At the time of sampling, only five participants were suspected or known to have contracted the disease. One participant displayed symptoms of an acute infection that was confirmed by a thick drop slide examination performed by the Health Surveillance Foundation FVS of Barcelos. Four participants were undergoing Benznidazole treatment for an acute infection that was reported after drinking natural patauá juice [56].

Blood procurement varied by requirement. A digital puncture was used to obtain whole blood for application in the immunochromatographic rapid test prototypes (two 10 µL samples). A brachial puncture was also performed to collect 5 mL of whole blood into a Vacutainer^®^ (BD, Franklin Lakes, NJ, USA) tube containing clotting factor. Serum was separated by centrifugation (2000× *g*, 10 min @ 25 °C), frozen at −20 °C, shipped to Manaus, AM, BR, for transport to Rio de Janeiro, RJ, on dry ice.

### 4.3. Cloning, Expression, and Purification of DxCruziV3

The amino acid sequence of DxCruziV3 was back translated and optimized for expression in *Escherichia coli* (Figure S2). The 3′ was extended to include a ribosome binding site and a 6xHis tag was added to the carboxy terminus. A gene fragment flanked by XbaI and XhoI was synthesized (Integrated DNA Technologies, Coralville, IA, USA) and inserted into the corresponding sites of pET28a. The final clone was confirmed by sequencing. Procedures to produce recombinant protein were pre-approved by the Internal Biosecurity Commissions of the Oswaldo Cruz Institute (No°5/5342017) and the Institute of Technology in Immunobiology (N° 006/2022) as a Class I biological risk.

For inclusion body-derived DxCruziV3 (V3_ib_), a confirmed clone of transformed *E. coli* BL21 (DE3) bacteria was cultured to log phase in 100 mL of Luria Broth (LB) with kanamycin (35 µg/mL) overnight at 37 °C with shaking (200 rpm). Next, 2 mL was inoculated into 100 mL of LB and cultured to an OD of 0.6 before induction with isopropyl-ß-D-thiogalactoside (IPTG; 1 mM). After 3 h, bacteria were collected by centrifugation (3500× *g*, 20 min @ 25 °C), resuspended in lysis buffer A (50 mM Na_2_HPO_4_, 500 mM NaCl; pH 7.4), and sonicated (amplitude 20 in pulses of 30 s with a 60 s pause for 2 min). Inclusion bodies were collected and washed twice with lysis buffer plus detergent (1% Triton X-100) by centrifugation (16,000× *g*, 20 min @ 4 °C). The final pellet was solubilized with 4 M urea in lysis buffer A, clarified (16,000× *g*, 20 min @ 4 °C), and applied to a pre-equilibrated 1 mL HisTrap^®^ HP column (Cytiva Latin America, São Paulo, Brazil) attached to an Äkta 10 chromatograph system (Cytiva Latin America). The column was washed with 10 column volumes (CV) of lysis buffer A with 4 M urea followed by 3 CV of lysis buffer A with 4 M urea and 22 mM imidazole. Bound V3_ib_ was eluted by a gradient of imidazole up to 500 mM. Elution fractions were analyzed by SDS-PAGE and Western blot (anti-6xHis antibody, ThermoFisher, São Paulo, Brazil) to identify fractions to combine for dialysis to reduce the molarity of urea to 1 M with PBS. The final concentration of V3_ib_ was determined by the Bradford method [57].

To produce soluble DxCruziV3 (sV3), a colony of the confirmed clone in *E. coli* BL21 (DE3) was cultured in 100 mL of LB with kanamycin (35 µg/mL) in a 37 °C incubator shaker at 220 rpm. After 16 h, the preculture was used to inoculate 2 L of LB with kanamycin in a bioreactor (Biostat^®^ B, Sartorius, Göttingen, Germany) maintained at 37 °C with an rpm of 500, an aeration rate of 1 VVM, and a pH of 6.8. At an OD_600_ of 0.6, expression was induced with IPTG (1 mM). After an additional 3 h, bacteria from 750 mL of culture were harvested by centrifugation and resuspended in 200 mL of lysis buffer B (50 mM sodium phosphate, 500 mM NaCl, 5% glycerol, and 20 mM imidazole; pH 7.5) containing cOmplete^®^, an EDTA-free protease inhibitor cocktail (Roche, Basil, Switzerland). Cells were disrupted by pressure (PandaPlus1000, GEA Niro Soavi, Parma, Italy) at 1000 bar and cycled for 30 min at 4 °C. The extract was centrifuged (16,000× *g*, 30 min @ 4 °C) and the supernatant containing sV3 was filtered (0.22 µm). A 5 mL HisTrap^®^ HP column (Cytiva Latin America) attached to an Äkta Pure chromatography system (Cytiva Latin America) was pre-equilibrated with 10 CV of lysis buffer B before loading the sample. It was washed with 10 CV of lysis buffer B followed by 5 CV of lysis buffer B with 50 mM imidazole and then 5 CV of lysis buffer containing 100 mM imidazole. Protein was eluted in a linear gradient range of imidazole in lysis buffer B from 100 to 500 mM. Fractions were analyzed by SDS-PAGE (12%) to pool those with sV3. Buffer exchange and sample concentration were performed using the Amicon^®^ Stirred system (Merck, São Paulo, Brazil) with a cellulose regenerated membrane (cutoff 5 kDa). The concentration of sV3 was calculated from its absorbance at 280 nm and the calculated molar absorption coefficient (30,370 M^−1^ cm^−1^). Purity was estimated by densitometry (GS-900, Bio-Rad, Hercules, CA, USA) of samples resolved by SDS-PAGE (12%).

### 4.4. Enzyme-Linked Immunosorbent Assays

Two commercial ELISA kits, Biolisa Chagas Recombinant (Bioclin, Belo Horizonte, MG, Brazil) and Chagatest recombinant v3.0 (Wiener Labs, Rosario, Argentina), were executed as directed by the manufacturer. In-house ELISA assays consisted of 96-well plates (Nunc™ MicroWell™, Rochester, NY, USA) sensitized overnight at 4 °C with 500 ng/well of V3_ib_ diluted into sodium carbonate–bicarbonate buffer (0.05 M, pH 9.6). Next, plates were washed 3× with PBS and blocked at 37 °C with 1X PBS-T (pH 7.4 with 0.05% Tween^®^ 20) with 5% powdered non-fat milk. After 1 h, buffer was removed and serological samples diluted, as indicated, in 100 µL of PBS-T with 1% powdered non-fat milk or the dilution series of the WHO International Biological Reference Standards, followed by another 1 h incubation at 37 °C. After three washes with PBS-T, goat anti-human IgG Fc-specific antibody conjugated to HRP (Cat # A0170, Sigma-Aldrich, St. Louis, MO, USA) was added at a dilution of 1:60,000 followed by another 1 h incubation at 37 °C. After three final washes with PBS-T, One Step—TMB Ultra (Scienco, Santa Catarina, Brazil) was added followed by an incubation at room temperature for 15 min in the dark. Enzymatic activity was stopped by the addition 0.5 M H_2_SO_4_. Absorbance was measured at 450 nm in an automated plate reader (Hidex Sense, Hidex, Turku, Finland).

### 4.5. Lateral Flow Immunochromatographic Assay Preparation

Lateral flow assays prepared with V3_ib_ (V3_ib_-LFA) began with the placement of a strip of nitrocellulose membrane to the center segment of a 3-segment adhesive card (30 cm × 5 cm) followed by the simultaneous application of lines of V3_ib_ (1 mg/mL at 1.5 µL/cm) and Protein A (1 mg/mL at 0.75 µL/cm) with an XYZ Platform Dispenser (HM3030, Shanghai Kinbio Tech, Shanghai, China). A glass fiber membrane previously treated in buffer (0.05 M Tris, 0.5% Casein, 1.0% PVP, 0.05% Tween^®^20, 0.02% sodium azide, pH 8.0) was impregnated with a spray of colloidal gold-labeled rabbit anti-human IgG antibody (ThermoFisher). After drying at 37 °C for 1 h, it was applied to the first segment of the adhesive card. A thick absorption pad was placed in the third segment. Strips (4 mm) were prepared on a programable strip cutter (ZQ2002, Shanghai Kinbio Tech) that were assembled in cassettes.

Lateral flow tests prepared with sV3 were manufactured by Bio-Manguinhos (Oswaldo Cruz Foundation, Rio de Janeiro, RJ, Brazil) under GMP conditions. The test strip consisted of a segment of nitrocellulose membrane immobilized on adhesive card. Solutions of sV3 (500 ug/mL, 0.85 µL/cm) and Protein A (50 mg/mL, 1 µL/cm) were applied as parallel lines by a lateral flow dispenser (Isoflow Dispenser, Imagene Technology Inc., Lebanon, NH, USA). Membranes were placed in an oven at 37 °C for 30 min to fix the proteins. A conjugation of a colloidal gold nanoparticle with protein A was deposited on a glass fiber, dried for 1 h in a 37 °C oven, and added to the adhesive card. After including an absorbent pad manually with the aid of a template, strips (5 mm) were obtained with a guillotine shear cutter (Matrix 2360, Kinematic, Sonora, CA, USA) that were fitted into plastic cassettes and closed with a compressor roller (AR3000, BioDot, Irvine, CA, USA).

### 4.6. Indirect Immunofluorescence

The indirect immunofluorescence assays (IFI Chagas, Bio-Manguinhos) were performed according to the included instructions.

### 4.7. Statistical Analysis

Students *t*-test analyses, ANOVA, and receiver operating characteristic (ROC) calculations were performed using Prism software (V8.0.2, GraphPad, Boston, MA, USA). Statistical significance was defined as a *p* value less than 0.05. The ROC analysis was used to define the cutoff value for reactive and non-reactive samples as well as to calculate the reactivity index (RI) as the division of an absorbance value divided by the cutoff (O.D./cutoff). Results within 10% of an RI of 1 were considered inconclusive.

## 5. Conclusions

Our multi-epitope protein DxCruziV3 represents a technological breakthrough for grouping a large number of epitopes into a single molecule. The experimental design and results suggest that as an immunological reagent, it addresses current test limitations for the diagnosis of chronic Chagas disease. The outcome is two-fold: a high-performance ELISA for improving the management of Chagas disease, and a qualified lateral flow assay for rapidly delivering actionable results. Their analytical sensitivities are greater than the recommendations by the WHO and their high specificities suggest that these assays would be refractory to co-infections. As a single recombinant protein produced in bacteria, its production on a larger scale could allow tests to be manufactured in greater quantities and at a more affordable cost. Together, the attributes of DxCruzi can facilitate diagnosis, seroepidemiology investigations, quality control, and improve studies in endemic areas.

## 6. Patents

A.M.D., S.G.D-S., and D.W.P. are listed as inventors on patents submitted in Brazil (BR10.2019.017792.6), USA (PCT/BR2020/05034 1), Europe (PCT: 26/06/2023), India (PCT: 26/06/2023), and China (PCT: 26/03/2023) filed by and under the administration of Oswaldo Cruz Foundation that may serve as a future source of funding.

## Figures and Tables

**Table 1:** Epitopes contained in DxCruziV3 and their position within Thermal Green Protein.

Epitope	Sequence	Insertion Site ^1^	Color ^2^	Protein Origin	Reference
1	KFAELLEQQKNAQFPGK	N-term	Red	KMP11	[26]
2	DSSAHSTPSTPA	50	Blue	SAPA	[27]
3	GDKPSPFGQAAAADK	114	Yellow	PEP-2	[28,29]
4	FGQAAAGDKPS	127	Magenta	TcCA-2	[30]
5	AEPKPAEPKS	153	Lt. Blue	TcD-2	[31,32]
6	TSSTPPSGTENKPAT	167	Cyan	TSSA	[28,33]
7	GTSEEGSRGGSSMPS	183	Salmon	TcLo1.2	[32]
8	SPFGQAAAGDK	244	Pink	B13	[29,34]
9	KAAIAPA	C-term	Purple	TcE	[32]
10	KQRAAETK	C-term	Orange	CRA	[35]

^1^ Based on the sequence in FPbase (https://www.fpbase.org/protein/thermostable-green-protein; accessed 3 July 2024). ^2^ Colorization of the sequence in the model presented in Figure 1.

**Table 2:** Results obtained by different assays. ^1^—In cases of divergence, additional assays were performed to determine a final call. ^2^—Whole blood was applied. ^3^—The number of tests in the first lot was insufficient to repeat all samples. ^4^—Second lot of sV3-LFA produced by Bio-Manguinhos.

Assay	Repetitions ^1^	Volume	Reactive	Non-Reactive
V3_ib_-LFA Field ^2^	1	10 µL	43	124
sV3-LFA Field ^2^	1	10 µL	43	124
V3_ib_-LFA Lab ^3^	0–1	5 µL	45	25
sV3-LFA Lab (Lot#1) ^3^	0–1	5 µL	44	123
sV3-LFA Lab (Lot #2) ^4^	1	5 µL	36	131
V3_ib_ ELISA (in-house)	3–8	0.5–2 µL	39	128
ELISA Wiener	2–3	10 µL	32	135
ELISA Bioclin	2	10 µL	61	106
Immunofluorescence Indirect	1–2	5 µL	48	119

**Table 3:** Performance of diagnostic assays for reactivity to chronic *T. cruzi* infections.

Assay	Non-Divergent (n = 150)		Final Call (n = 167)	
	Sensitivity	Specificity	Sensitivity	Specificity
Field V3_ib_ LFA	100%	100%	95.2%	97.6%
Field sV3 LFA	100%	100%	90.5%	96.0%
V3_ib_-ELISA	87.5%	98.3%	87.5%	97.6%
ELISA Wiener	71.9%	97.5%	64.3%	96.0%
ELISA Bioclin	90.6%	79.7%	83.3%	79.2%
Immunofluorescence	93.8%	88.1%	76.2%	87.2%

## Data Availability

Dataset available on request from the authors.

## Supplementary Materials

The following supporting information can be downloaded at: https://www.mdpi.com/article/10.3390/ijms25189811/s1.
