# Supplementary material for: A Multi-Epitope Protein for High-Performance Serodiagnosis of Chronic Chagas Disease in ELISA and Lateral Flow Platforms
Source: Int J Mol Sci. 2024 Sep 11;25(18):9811. doi: 10.3390/ijms25189811 (PMC11432030; doi:10.3390/ijms25189811)
Supplement: Supplementary file 1 [file ijms-25-09811-s001.zip › 20240902_DxCruziV3_Suppl.pdf]

Table S1: Quality control of sV3-LFA produced by Bio-Manguinhos.

sV3-LFA	Chagas disease	
	Positive	Negative
Positives (n=50)	48	2
Negatives (n=105)	0	105
**Sensitivity**	**96%**	
**Specificity**	**100%**	

Table S2: Test sites of the V3-LFA prototypes in the municipality of Barcelos, AM, BR.

	V3ib LFA		sV3 LFA	
Community	Reactive	Non-Reactive	Reactive	Non-Reactive
Aku Aku	0	7	0	7
*Aracá	1	0	1	0
Cumaru	4	11	4	11
**Demeni	0	1	0	1
*Floresta	0	3	0	3
*Lesbão	0	1	0	1
*Manacauaca	0	1	0	1
Mariuá	12	24	12	24
*Nova Esperança	0	9	0	9
Nova Jerusalém	19	33	20	32
*Pedro Segundo	0	1	0	1
Piloto	6	16	5	17
*Romão	0	1	0	1
Tapera	1	10	1	10
Telheiro	0	2	0	2
*Unini	0	4	0	4
Total	43	124	43	124

*Temporary residence on boats on the banks of the Rio Negro during the 2022 Brazilian election period.
**Confirmed to be an acute infection with *T. cruzi* by a thick drop blood smear.

Table S3: Sex, age, and rapid test results of participants with percent reactive per group.

LFA Result	Biological gender		Age (years)			
	Male	Female	4-17	18-40	>40	Not Informed
Reactive	32 (31%)	11 (17%)	2 (29%)	4 (6%)	38 (43%)	1 (50%)
Non-reactive	70	54	5	65	51	1
Total	102	65	7	69	89	2

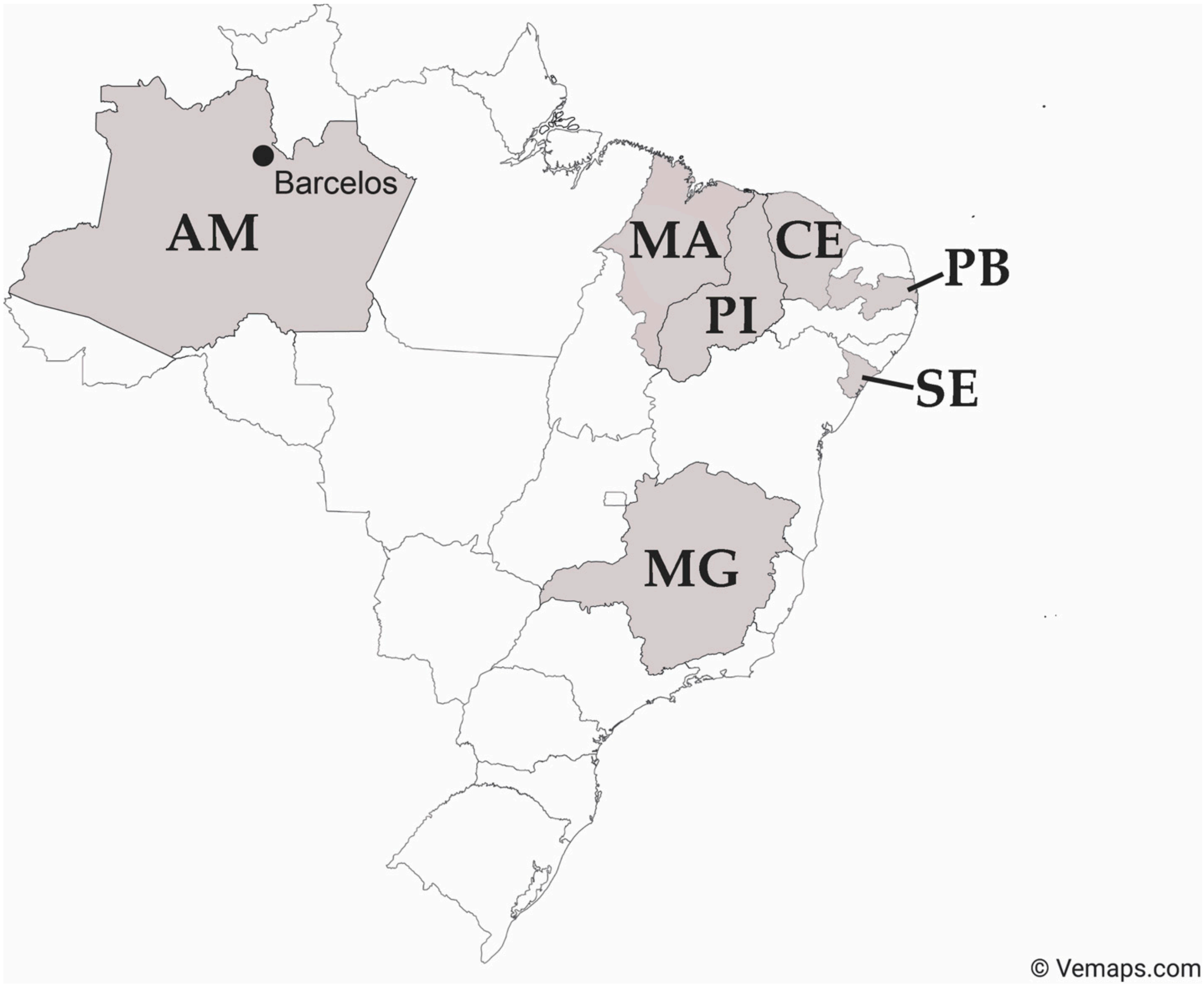

Figure S1: Origins of patient serum used in the study. A map of Brazil showing the states of Amazonas (AM), Maranhão (MA), Piauí (PI), Ceará (CE), Paraíba (PB), Sergipe (SE), and Minas Geiras (MG).

```
   1 GGATCCAATAATTTTGTTTAACTTTAAGAAGGAGATATACC

   1 M   G   A   H   A   S   V   I   K   F   A   E   L   L   E   Q   Q   K   N   A
  42 ATGGGTGCGCACGCAAGCGTGATTAAGTTTGCGGAACTTTTAGAACAGCAGAAAAACGCA
  21 Q   F   P   G   K   P   E   M   K   I   K   L   R   M   E   G   A   V   N   G
 102 CAGTTCCCGGGGAAACCCGAAATGAAAATCAAACTTCGCATGGAGGGGGCGGTAAACGGC
  41 H   K   F   V   I   E   G   E   G   I   G   K   P   Y   E   G   T   Q   T   L
 162 CACAAGTTTGTTATCGAGGGGGAAGGTATCGGAAAGCCTTACGAGGGAACGCAGACTTTG
  61 D   L   T   V   E   E   D   S   S   A   H   S   T   P   S   T   P   A   Y   D
 222 GACTTAACAGTAGAGGAAGACTCATCTGCACATTCAACCCCGTCTACTCCGGCATATGAT
  81 I   L   T   P   A   F   Q   Y   G   N   R   A   F   T   K   Y   P   E   D   I
 282 ATTTTAACTCCTGCGTTTCAATATGGGAACCGTGCATTTACTAAATACCCAGAGGACATC
 101 P   D   Y   F   K   Q   A   F   P   E   G   Y   S   W   E   R   S   M   T   Y
 342 CCTGATTATTTCAAACAGGCTTTTCCGGAAGGTTACTCATGGGAGCGCTCTATGACATAT
 121 E   D   Q   G   I   C   I   A   T   S   D   I   T   M   E   G   D   K   P   S
 402 GAAGATCAAGGAATCTGTATTGCCACTTCGGACATCACCATGGAGGGAGACAAGCCGAGC
 141 P   F   G   Q   A   A   A   A   D   K   C   F   F   Y   E   I   R   F   D   G
 462 CCATTTGGTCAAGCAGCTGCCGCAGATAAATGTTTTTTCTATGAAATTCGTTTCGACGGG
 161 T   F   G   Q   A   A   A   G   D   K   P   S   T   L   K   W   E   P   S   T
 522 ACATTTGGACAAGCGGCTGCAGGCGACAAGCCTAGCACGTTGAAGTGGGAGCCAAGCACC
 181 E   K   M   Y   V   E   A   E   P   K   P   A   E   P   K   S   V   L   K   G
 582 GAAAAGATGTACGTTGAGGCTGAACCGAAGCCAGCGGAGCCGAAATCAGTCTTAAAGGGG
 201 D   V   E   M   A   L   L   L   T   S   S   T   P   P   S   G   T   E   N   K
 642 GATGTTGAAATGGCTTTGCTTCTGACGAGCAGCACGCCACCAAGTGGCACAGAAAACAAA
 221 P   A   T   G   H   Y   R   C   D   F   K   T   T   Y   K   A   G   T   S   E
 702 CCCGCCACAGGACATTATCGCTGCGATTTTAAGACTACATATAAGGCTGGTACCTCTGAG
 241 E   G   S   R   G   G   S   S   M   P   S   H   E   V   D   H   R   I   E   I
 762 GAGGGGTCTCGCGGAGGTAGTAGCATGCCGTCACACGAGGTTGACCACCGCATTGAGATC
 261 L   S   H   S   P   F   G   Q   A   A   A   G   D   K   K   V   R   L   Y   E
 822 TTATCTCACTCCCCTTTTGGTCAGGCTGCAGCTGGGGATAAGAAGGTGCGTCTTTATGAG
 281 H   A   E   A   R   Y   S   G   G   G   S   G   K   A   A   I   A   P   A   G
 882 CACGCGGAGGCCCGTTACTCTGGTGGAGGCAGTGGGAAAGCGGCAATTGCCCCCGCAGGC
 301 G   A   S   G   K   Q   R   A   A   E   A   T   K   P   I   P   N   P   L   L
 942 GGCGCGTCAGGGAAACAACGCGCCGCTGAGGCGACGAAACCGATCCCGAACCCGCTGTTG
 321 G   L   D   S   T   H   H   H   H   H   H   K   K   *
1002 GGACTTGACAGTACCCACCATCATCACCACCACAAGAAATAGCTCGAG
```

Figure S2: DxCruziV3. Amino acid sequence with the back translated DNA coding sequence along with the 5′ extension as well as the restriction sites of XbaI and XhoI (underlined) for insertion into pET28a

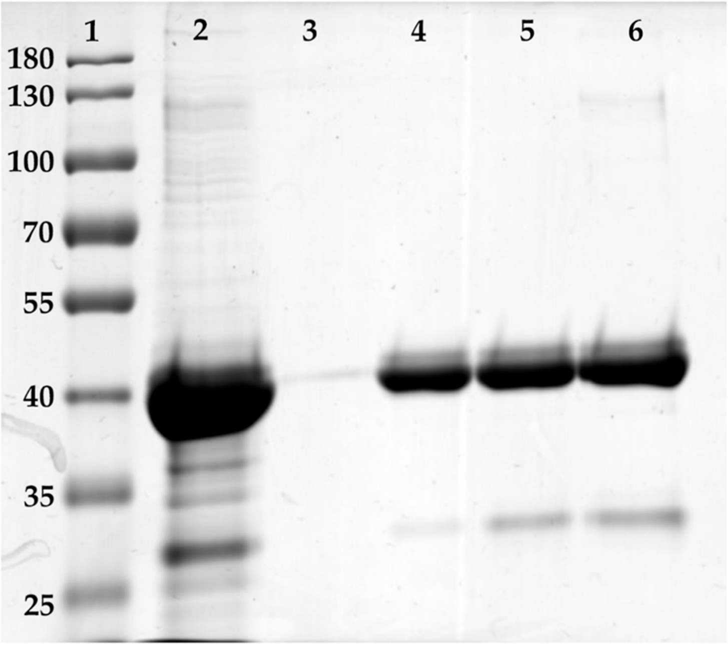

Figure S3: Preparation of DxCruziV3 from inclusion bodies. An SDS-PAGE (12%) separation of equal sample volumes (10 µl) from a representative purification of DxCruziV3 (≅ 36 kDa) with HisTrap™ column on an Äkta chromatograph system. Lane 1- Molecular weight standard (kDa); 2- Solubilized inclusion body fraction (load) 3- Flow through; 4- Elution Fraction 20; 5- Elution Fraction 24; 6- Elution Fraction 25.

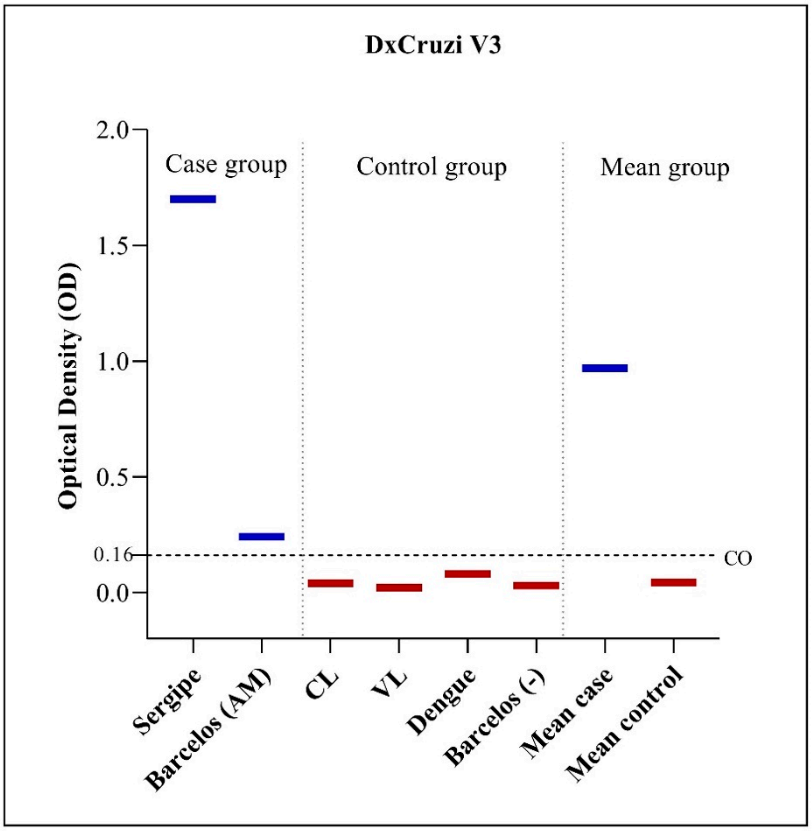

Figure S4: Reactivity of serum pools in a V3ib ELISA. The development of the V3ib lateral flow assay employed serum pools assembled from patients with chronic Chagas disease and a high (Sergipe) or low (Barcelos) antibody titer, cutaneous leishmaniasis (CL), visceral leishmaniasis (VL), dengue, or negative for Chagas disease from Barcelos (Barcelos (-)). Each pool combined 10 individual serological samples and applied to a V3ib-ELISA at a dilution of 1:200. CO = cutoff.

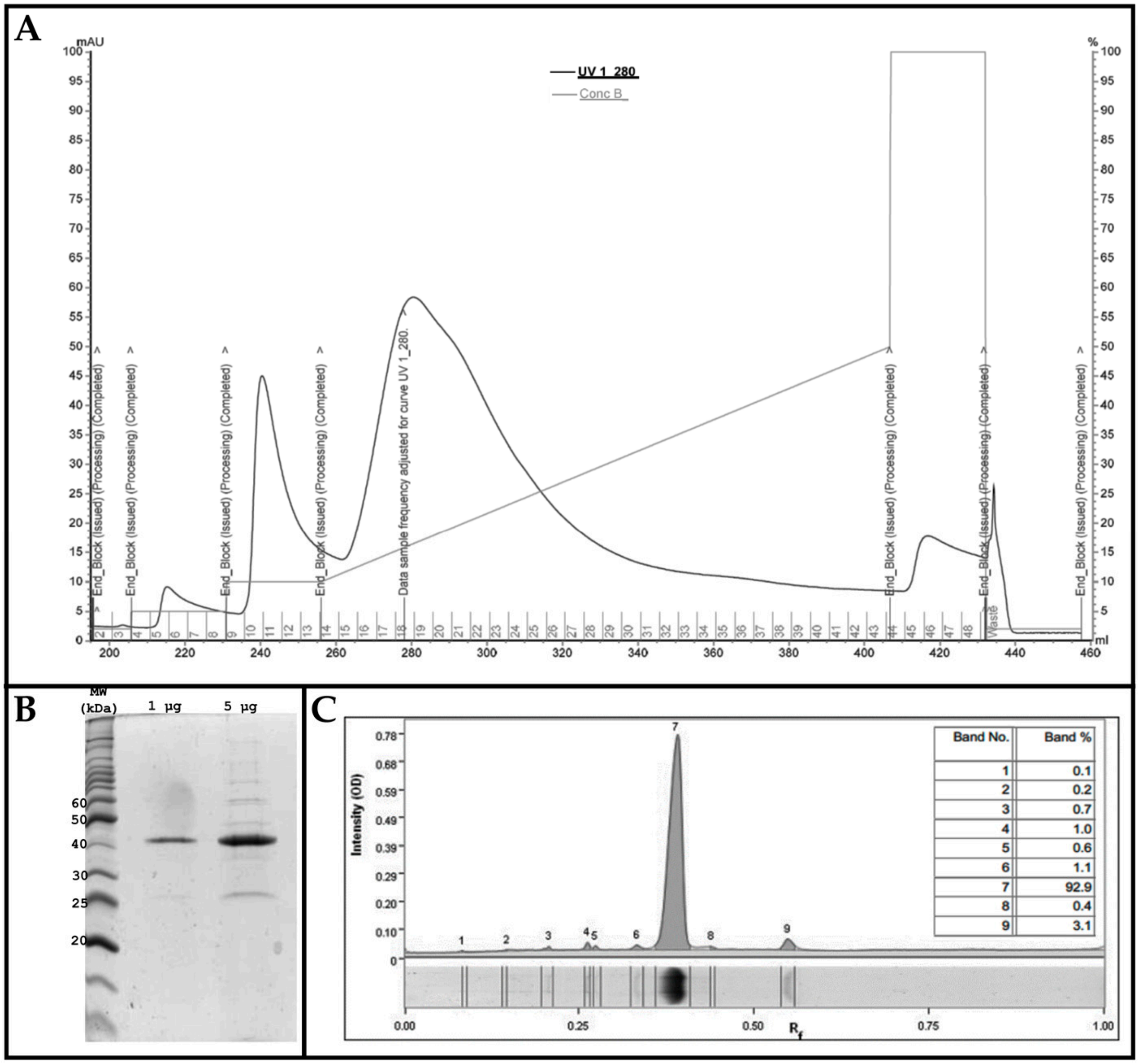

Figure S5: Preparation of soluble DxCruziV3. (a) A chromatograph of the metal (Ni-NTA) affinity purification of DxCruziV3 with the measured absorbance units (280 nm; left y-axis) of the eluate and the percentage of 500 mM imidazole (right y-axis). (b) An SDS-PAGE (12%) analysis of the pool of fractions 16-41 at 1 µg and 5 µg total protein in comparison to molecular weight standards. (c) A densitometry of the lane with 5 µg of protein with the percentage of signal for each protein band.

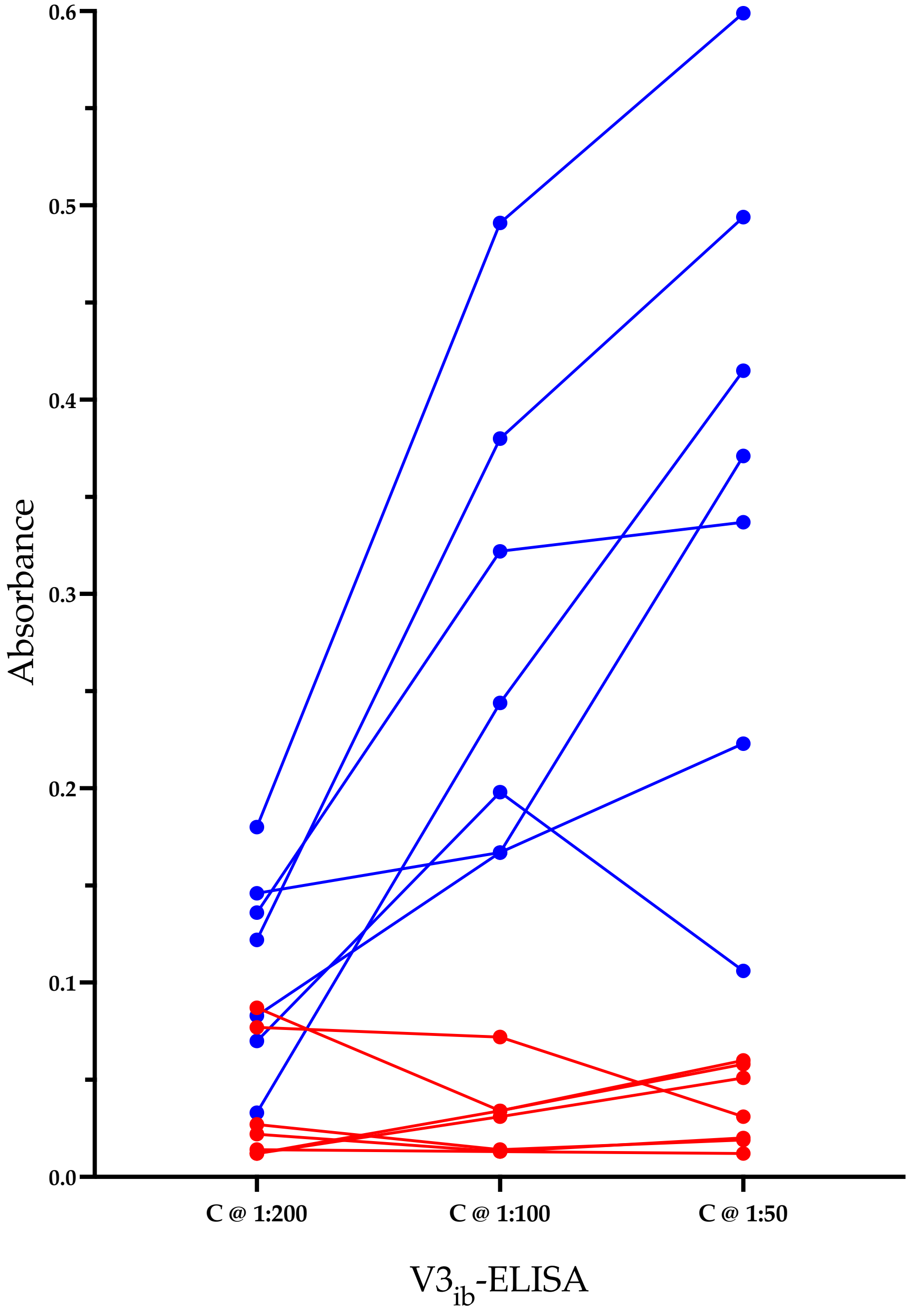

Figure S6: Serial dilution analysis of antibody titer in select patient samples with divergent serodiagnostic results. Serum dilutions of 1:200, 1:100, and 1:50 were screened by $V3_{ib}$ ELISA that either showed little difference in absorbance values (non-reactive, red) or increasing values (reactive, blue).
